# It’s a competitive business

**DOI:** 10.7554/eLife.96304

**Published:** 2024-02-23

**Authors:** Masaaki Sokabe, Christopher S Fraser

**Affiliations:** 1 Department of Molecular and Cellular Biology, College of Biological Sciences, University of California, Davis Davis United States

**Keywords:** translation, ribosomes, purified reconstituted systems, molecular biology, mRNA, Other

## Abstract

A new in vitro system called Rec-Seq sheds light on how mRNA molecules compete for the machinery that translates their genetic sequence into proteins.

**Related research article** Zhou F, Bocetti JM, Hou M, Qin D, Hinnebusch AG, Lorsch JR. 2024. Transcriptome-wide analysis of the function of Ded1 in translation preinitiation complex assembly in a reconstituted in vitro system. *eLife* **13**:RP93255. doi: 10.7554/eLife.93255.

Cells respond to changes in their environment – such as stress, pathogens and growth signals – by altering the proteins they make. The last stage of producing a protein involves a ribosome reading a molecule of mRNA and translating its genetic code into a string of amino acids. However, translating the wrong mRNA at the wrong time can lead to diseases such as cancer or mental illness. Moreover, it is still not fully understood how a ribosome ‘knows’ which mRNA to translate.

For translation to begin, two things need to happen: (i) the correct mRNA strand needs to be recruited to a ribosome; and (ii) the ribosome needs to know where along the mRNA strand it should begin translating. These decisions are highly regulated and depend on the genetic sequence of each mRNA and the availability and activity of proteins called initiation factors (Merrick and Pavitt, 2018).

To better understand how translation is regulated, biochemists have built purified reconstituted systems which recreate translation in vitro using individual components purified from cells (Pisarev et al., 2007). While these systems allow researchers to study translation independently from other mechanisms required for protein production, they can only analyze one mRNA at a time. This is a significant problem because thousands of different mRNAs can be present in a cell, and they compete with each other for a limited number of initiation factors and ribosomes. Now, in eLife, Jon Lorsch, Alan Hinnebusch and colleagues from the National Institutes of Health – including Fujin Zhou as first author – report a new purified reconstituted system made with components from yeast (*Saccharomyces cerevisiae*) which can incorporate all mRNAs of a cell (Zhou et al., 2024).

The team combined its purified reconstituted system with a deep sequencing method known as Ribo-Seq, which provides a global snapshot of where translating ribosomes are precisely positioned on all mRNAs in a cell (Ingolia et al., 2013). This approach, named Rec-Seq, means that a purified reconstituted system can now be used to study how individual components regulate which mRNAs are recruited to ribosomes and the location of the start site for translation (Figure 1).

**Figure 1.**
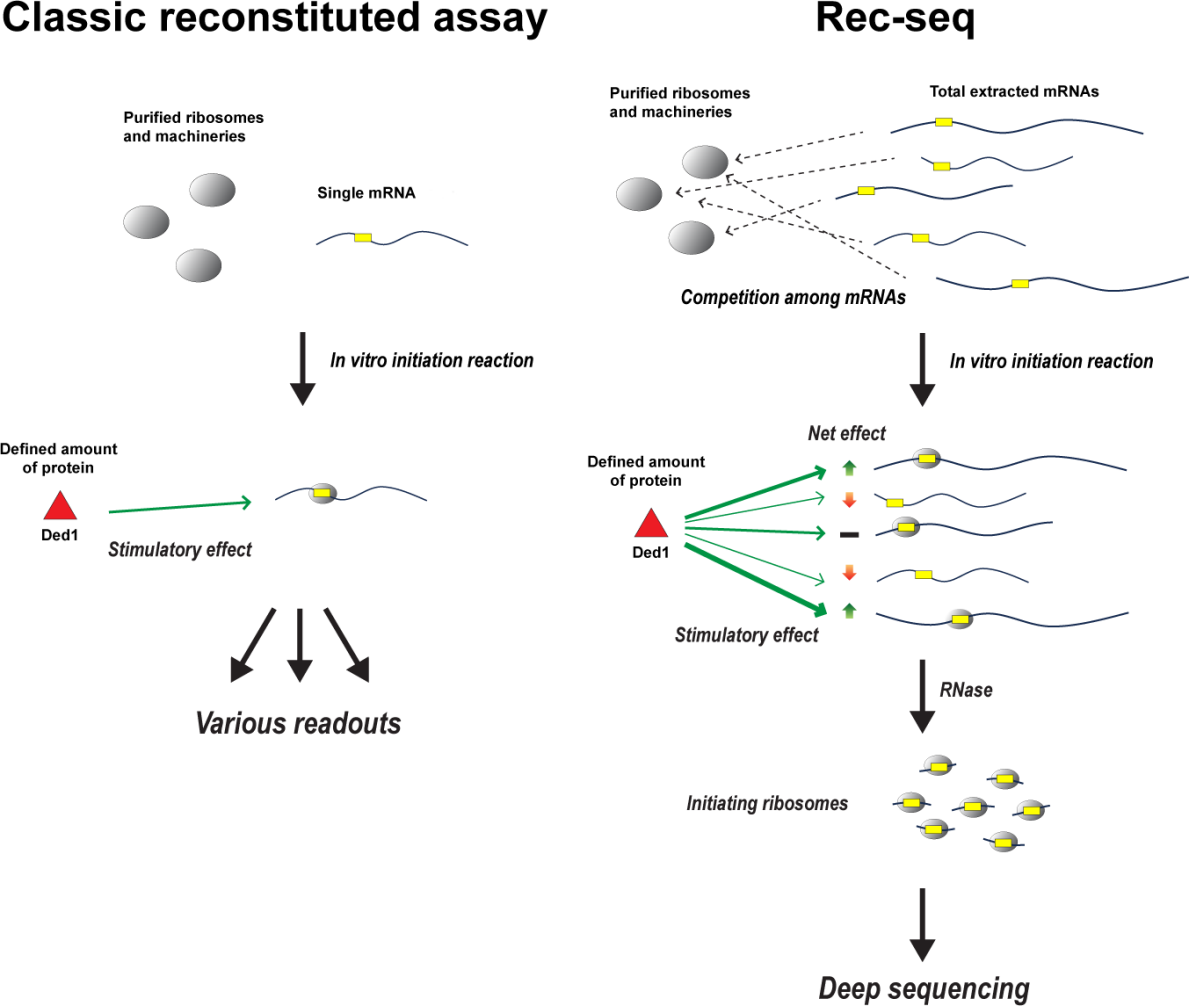
How Rec-Seq compares to classic purified reconstituted systems. In classic reconstituted assays (left), components involved in translation – such as ribosomes and initiation factors – are purified from cells and combined with a single molecule of mRNA (yellow rectangular box indicates the start site of translation). Translation is then initiated in vitro to see how these components, such as the enzyme Ded1 (red triangle), impact the readout produced from the mRNA strand. Zhou et al. have expanded this approach to create a new purified reconstituted system called Rec-Seq that can interact with all mRNAs extracted from a cell (right), mimicking the cellular environment where thousands of different mRNAs compete for a limited number of ribosomes and initiation factors. Deep sequencing is then applied to the mRNA molecules, which have been broken down by the enzyme RNase, to reveal how individual components like Ded1 impact translation. For instance, which mRNA strands are translated more (green up arrow), less (red down arrow), or are unaffected (black bar) when Ded1 is present, and how Ded1 impacts the start site where ribosomes begin translating from.

Previous in vivo experiments on yeast cells using the Ribo-Seq method found that the helicase enzyme Ded1 (known as DDX3X in humans) promotes recruitment to the ribosome and determines where translation will be initiated for over 600 mRNAs (Sen et al., 2015). However, Ded1 is involved in many other RNA processes, including the formation of ribosomes, stress granules, and P-bodies (Sharma and Jankowsky, 2014). Ded1-dependent changes in these processes may affect translation, making it difficult to interpret whether Ded1 directly or indirectly regulates translation. Moreover, changing the amount of any component involved in translation could down-regulate (or up-regulate) the level of other proteins that may impact this process.

Lorsch and co-workers have previously shown that the Ded1 helicase enzyme has a direct role in promoting the translation initiation of a handful of the mRNAs identified by Ribo-Seq (Gupta et al., 2018). However, their new Rec-Seq system revealed over 1,000 mRNAs require Ded1 to be efficiently recruited to ribosomes when all mRNAs are present and competing with one another. Consistent with Ribo-Seq data, the untranslated end of these mRNAs was relatively long and highly structured. This suggests that Ded1 has a role in unwinding mRNA molecules to promote their recruitment to ribosomes and to expose their start site for translation.

Zhou et al. also varied the amount of another helicase enzyme, eIF4A. Rec-Seq analysis showed that essentially all mRNAs required this enzyme to be recruited to ribosomes regardless of the structural complexity of their untranslated end, consistent with previous findings (Yourik et al., 2017). Together, this work highlights that the helicase enzymes Ded1 and elF4A have distinct regulatory roles in translation.

In the future, it will be interesting to add more complexity to the Rec-Seq system so that it can capture other regulatory processes, such as the regulation of translation by upstream open reading frames or the role of other proteins that bind to specific mRNAs. The method could also be calibrated using available Ribo-Seq data to remove potential artifacts and biases that are inherent to reconstituted systems.

Nevertheless, the current Rec-Seq approach developed by Zhou et al. provides an excellent purified reconstituted system for studying what happens during translation when all the mRNAs of a cell are able to compete for ribosomes. This breakthrough will allow scientists to better understand how translation works in a ‘typical’ cellular environment. It may also lead to the discovery of delicate, yet physiologically important, regulatory mechanisms that cannot be readily detected using traditional reconstituted systems.
